# Multiple Functions of Phosphoinositide-3 Kinase Enhancer (PIKE)

**DOI:** 10.1100/tsw.2010.64

**Published:** 2010-04-13

**Authors:** Chi Bun Chan, Keqiang Ye

**Affiliations:** Department of Pathology and Laboratory Medicine, Emory University School of Medicine, Atlanta, GA, USA

**Keywords:** Akt, apoptosis, cancer, PIKE, neuron, phosphoinositide 3-kinase

## Abstract

GTP-binding proteins are the molecular switches of numerous cellular functions, including migration, proliferation, and differentiation. In the past 10 years, we have characterized a novel class of GTPases called phosphoinositide-3 kinase enhancer (PIKE) that interacts with PI3K/Akt. In neurons, PIKE is involved in the protective mechanisms against neuroexcitotoxic insults by linking various receptors, such as mGluR1 and Unc5H2, to the PI3K cascade. Interestingly, the *PIKE* gene (CENTG1) is also amplified in a variety of human cancers and enhances Akt activity, leading to their resistance against apoptosis and invasion. It has also been demonstrated that PIKE is involved in endosome formation and nuclear protein accumulation. Thus, PIKE possesses multitudinous biological functions rather than solely acting as an apoptosis inhibitor. In this review, we will update our current knowledge of the role of PIKE in various cellular activities. Moreover, the functions of PIKE in peripheral tissues as revealed by the unique phenotypes in PIKE knock-out mice will be discussed as well.

## INTRODUCTION

Phosphoinositide 3-kinase enhancer (PIKE) is a group of GTP-binding proteins that possess GTPase activity. Three isoforms of PIKE, which are named PIKE-S, PIKE-L, and PIKE-A, have been identified. Structural analysis of PIKE with other GTP-binding proteins suggested that they belong to a subclass of the centaurin family (centaurin γ1)[1]. Although it has not been experimentally confirmed, it is believed that the three PIKE isoforms are generated by alternative splicing and differential promoter usage of the *CENTG1* gene[2]. Structurally, PIKE-L is the longest isoform among the three family members, containing a proline rich N-terminus, a central GTP-binding domain, a pleckstrin homology (PH) domain, an Arf-GAP–like sequence, and two ankyrin repeats[3]. Compared to PIKE-L, PIKE-S lacks the Arf-GAP–like domain and ankyrin repeats in the C-terminus[2]. PIKE-A is the C-terminal truncate of PIKE-L in which the N-terminal proline-rich domain is replaced with a unique short peptide of 72 amino acids[4,5]. Since the first report of PIKE-S as a novel nuclear PI3K activator in response to NGF stimulation[2], the functions of PIKE proteins have been drastically expanded in different areas of cellular functions, including antiapoptosis, tumor transformation, membrane trafficking, cell cycle progression, and nuclear transportation. In this review, we will discuss recent advances in our understanding of the roles of PIKE in different aspects of cellular activities. Moreover, we will briefly introduce some findings on the novel functions of PIKE using a *PIKE* knock-out mice model.

## FUNCTION OF PIKE DURING APOPTOSIS

Phosphatidylinositol lipids are important molecules in a multitude of cellular responses from cell proliferation to inflammation[6]. The lipid kinase phosphoinositide 3-kinase (PI3K) is one of the key molecules in regulating the production of various 3' phosphorylated inositides in response to various stimuli[7]. In particular, the nuclear PI3K contributes significantly to protect the cells from apoptosis as activation of nuclear PI3K by growth factor is sufficient to protect the nucleus from apoptotic damage[8]. In addition to the canonical view that the enzyme localizes solely on the cell membrane to couple the production of phosphoinositide to receptor activation, it has now been generally accepted that a functional distinct set of PI3K presents in the nucleus and utilizes an independent regulatory system[9]. Nevertheless, the regulatory mechanism on nuclear phosphoinositol metabolism remains poorly characterized.

The first function of PIKE was demonstrated in 2000, when we reported that PIKE-S is a nuclear GTPase in response to nerve growth factor (NGF) stimulation to activate nuclear PI3K in PC12 cells[2] (Fig. 1). It is well recognized that growth factors like NGF are able to induce PI3K activation in the nucleus, but the mechanism remains unknown[10,11]. We showed that PIKE-S bound to both p85 and p110 subunits of the nuclear PI3K in a GTPase-dependent manner. Association of PIKE-S and PI3K leads to an augmentation of its kinase activity. Importantly, activation of PIKE-S and its subsequent activation of PI3K are abolished if PIKE-S GTPase is artificially mutated. Association of PIKE-S and PI3K is thus necessary for NGF to trigger cyclin D1 expression[12]. The activation of PIKE-S could be attenuated by protein 4.1N[2]. Protein 4.1N is a neuronal-specific isoform of 4.1 family proteins, and is involved in multiple functions ranging from cytoskeleton networking to RNA processing[13]. Hours after NGF stimulation, 4.1N is translocated into the nucleus[14], where it interacts with PIKE-S and prevents it from PI3K association. The PI3K-enhancing activity of PIKE-S is thus attenuated, leading to a reduction of nuclear PI3K activity. These studies demonstrate that PIKE-S is a molecular switch that is responsible to convert the mitogenic signal of NGF in the nucleus.

By a combination of database searches and cDNA library screening, a PIKE-S isoform, now designated as PIKE-L, was identified in the central nervous system[3]. Different from PIKE-S, which is nuclear specific, PIKE-L localizes in both the cytoplasmic and nuclear compartment of neurons. Within the cytoplasm, it was found that PIKE-L associated with the isoform 1c of Homer proteins (Homer 1c) (Fig. 1). Homer proteins are a family of scaffold proteins residing at postsynaptic densities (PSDs), where they link the metabotropic glutamate receptor mGluRI with other PSD proteins, such as Shank and Ins(1,4,5)P3 receptors[15]. Since Homer 1c is well known as an adaptor to mGluRI[16], it is reasonable to infer that a molecular complex containing mGluR/Homer/PIKE-L exists in the neuronal membrane. This hypothesis was confirmed by a series of biochemical and immunoprecipitation analyses. Indeed, the formation of the mGluR/Homer/PIKE-L is triggered by glutamate receptor activation with its specific ligand quisqualate, and PIKE-L serves as a linkage between mGluRI and PI3K. These results provide a molecular mechanism for the antiapoptotic function of glutamate in neurons[17]. It is suggested that activation of mGluRI by its specific ligand triggers the interaction between PIKE-L, Homer 1c, and mGluRI. Consequently, PI3K is activated, leading to an initiation of antiapoptotic pathways, such as Akt activation[18]. This notion is further supported by the results that neurons overexpressing the PIKE-L mutant, which is unable to associate with Homer 1c, are insensitive to quisqualate-induced neuroprotection[3].

The antiapoptotic function of PIKE-L in neurons is further demonstrated by a recent study on its role in netrin signaling[19] (Fig. 1). Netrins are a family of secreted proteins that govern the migration of neurons and growth clones during neuronal development. Moreover, netrins are necessary to maintain the survival of developing neurons, as neurons that grow out of the specific regions of netrin expression will undergo apoptosis[20]. To perform their functions, netrins interact with specific receptors DCC (deleted

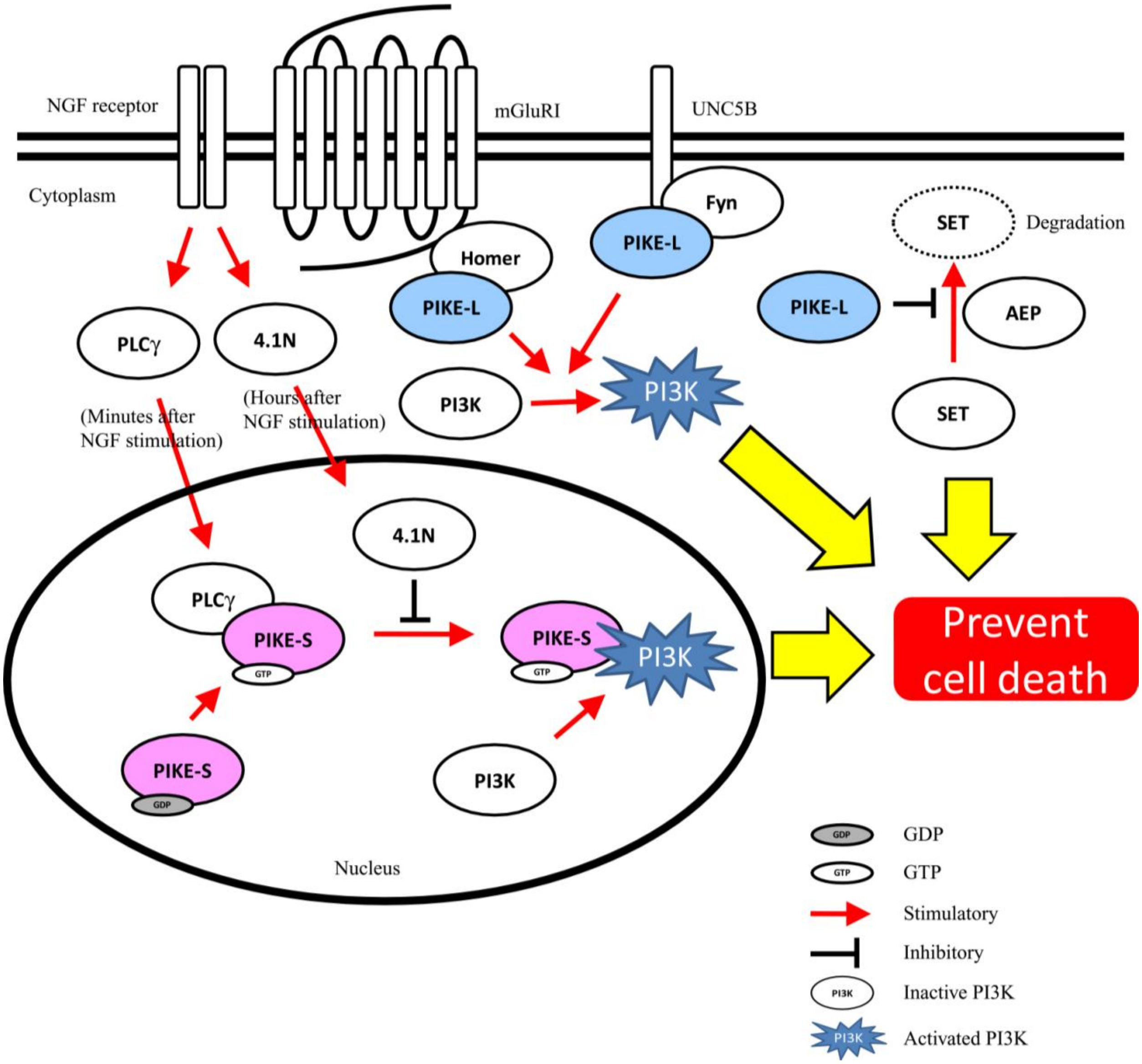

**FIGURE 1.** Mechanisms of PIKE-L and PIKE-S in inhibiting apoptosis.

in colorectal cancer) or UNC5[21,22]. Netrins trigger multiple signaling cascades, including raising cytosolic cAMP and $Ca^{2+}$[23], initiation of the MAPK pathway[24], FAK and Fyn activations[25], and inducing PI3K activities[26]. However, it is unknown which receptor(s) is(are) responsible for activating the particular signaling pathway. In an attempt to search for the interaction partner of PIKE-L, we found that PIKE-L specifically associated with the C-terminus of UNC5B by yeast-two-hybrid screening[19]. The formation of the neuronal UNC5B/PIKE-L complex is modulated by netrin-1 stimulation in a GTPase-dependent manner. Moreover, the association between PIKE-L and UNC5B is fyn dependent, that fyn phosphorylation on PIKE-L is a prerequisite for their interaction. Indeed, Fyn is a cytoplasmic kinase that could be activated by netrin[25], suggesting a functional coupling between UNC5B, fyn, and PIKE-L during netrin stimulation. Similar to PIKE-L/mGluRI interaction, PIKE-L is necessary for netrin to activate the neuronal PI3K pathway and maintains its neuroprotection ability. Nevertheless, it remains unexplored if this pathway is necessary for netrin-1 to carry out its function in axonal guidance.

In addition to linking various receptors to the cytosolic PI3K cascade, PIKE-L exerts its neuroprotective role via direct inhibition of apoptotic machinery. PIKE-L interacts with the DNase inhibitor SET and protects it from proteolytic cleavage[27] (Fig. 1). As such, the inhibitory activity of SET is preserved, thus preventing the genome from undergoing apoptotic DNA damage[28]. Since SET is cleaved by protease asparaginyl endopeptidase (AEP) during neurotoxic insults, when AEP is activated by the decreased intracellular pH[27], the degradation of SET will be more severe if the protection from PIKE is missing. In support of this hypothesis, SET cleavage is greatly enhanced in PIKE-depleted neurons after kainic acid treatment, suggesting that PIKE-L is necessary in order to maintain SET integrity by protecting its cleavage from AEP. Interestingly, depletion of PIKE-L also induced caspase 3 activation in the absence of neurotoxic insult[27]. Similarly, we observed elevated caspase 3 activation in the brain of PIKE knock-out (*PIKE -/-*) mice, which strongly suggests that PIKE is necessary for preventing the neurons from undergoing spontaneous apoptosis (unpublished observations).

## PIKE AS A PROTO-ONCOGENE

A proto-oncogene is defined as a gene that contributes to cancer formation when altered by mutation or overexpression. When the activity of a proto-oncogene is altered, usually up-regulated, the cells will proliferate in an uncontrolled manner. PIKE-A is the only isoform in the PIKE family that has a broad expression pattern[29,30,31]. As a matter of fact, enhanced expression of PIKE-A has also been detected in numerous tumors. Using cDNA array screening, it was found that PIKE-A expression was strongly elevated in a variety of human tumors derived from breast, ovary, colon, stomach, lung, kidney, prostate, uterus, cervix, rectum, testis, and skin[32]. Moreover, it has been reported that amplification of chromosome 12q13-q15, where *CENTG1* locates, was frequently observed in sarcomas and brain tumors[33,34,35]. In agreement with this, we reported that PIKE-A was expressed in glioblastomas, anaplastic astrocytoma, sarcoma, and neuroblastoma[4,5]. Knobbe et al. further examined the expression of PIKE-A in glioblastoma samples and found that about 12% of glioblastomas studied display *CENTG1* amplification. They also showed a more than twofold increase of PIKE-A transcript in about 93% of tumor samples without *CENTG1* amplification[36]. These results suggest a strong correlation between PIKE-A expression and tumor formation. In addition, we have demonstrated that PIKE-A overexpression was sufficient to transform NIH3T3 cells and enhance U87MG, a glioblastoma cell line without CDK4 amplicon and with modest PIKE-A expression, proliferation, and invasion[32]. Therefore, PIKE-A satisfies the criterion of a proto-oncogene. Accordingly, overexpression of PIKE-A is found in multiple forms of tumor and overexpression of PIKE-A in normal cells leads to uncontrollable proliferation.

The exact molecular mechanism of how PIKE-A promotes cell malignancy is not fully understood. Glioblastomas with high PIKE-A overexpression are more resistant to apoptotic stimulation[4]. Since PIKE-L and PIKE-S potentiate the activity of PI3K, it would be logical to infer that PIKE-A might exert the antiapoptotic function through activating PI3K. To our surprise, PIKE-A does not associate with PI3K, probably because of the lack of the proline-rich N-terminus found in other PIKE isoforms. Instead, PIKE-A interacts with activated Akt, the downstream effector of PI3K, through its GTPase domain in a GTP-dependent manner[5] (Fig. 2). The association with PIKE-A potentiates Akt's kinase activity, thus facilitating the stimulatory function of Akt in cell invasion and proliferation[5]. The detailed mechanism of how PIKE-A provokes the Akt activity yet remains to be explored; it is hypothesized that PIKE-A preferentially binds the phosphorylated C-terminal of Akt, where both $Thr^{308}$ and $Ser^{473}$ residues are located[37,38], masks the phosphorylated region, and shields it away from protein phosphatase, thus preventing Akt from inactivation.

Since tumor cells with high PIKE-A expression also possess high invasion, the identification of how PIKE-A mediates the process might be beneficial for preventing metastasis. By directly associating with focal adhesion kinase (FAK) and enhancing its activities, PIKE-A is reported to be involved in the disassembly of focal adhesion, a macromolecular structure that is responsible for cell anchorage to the extracellular matrix and transduces various stimuli from the environment to various cell compartments[39]

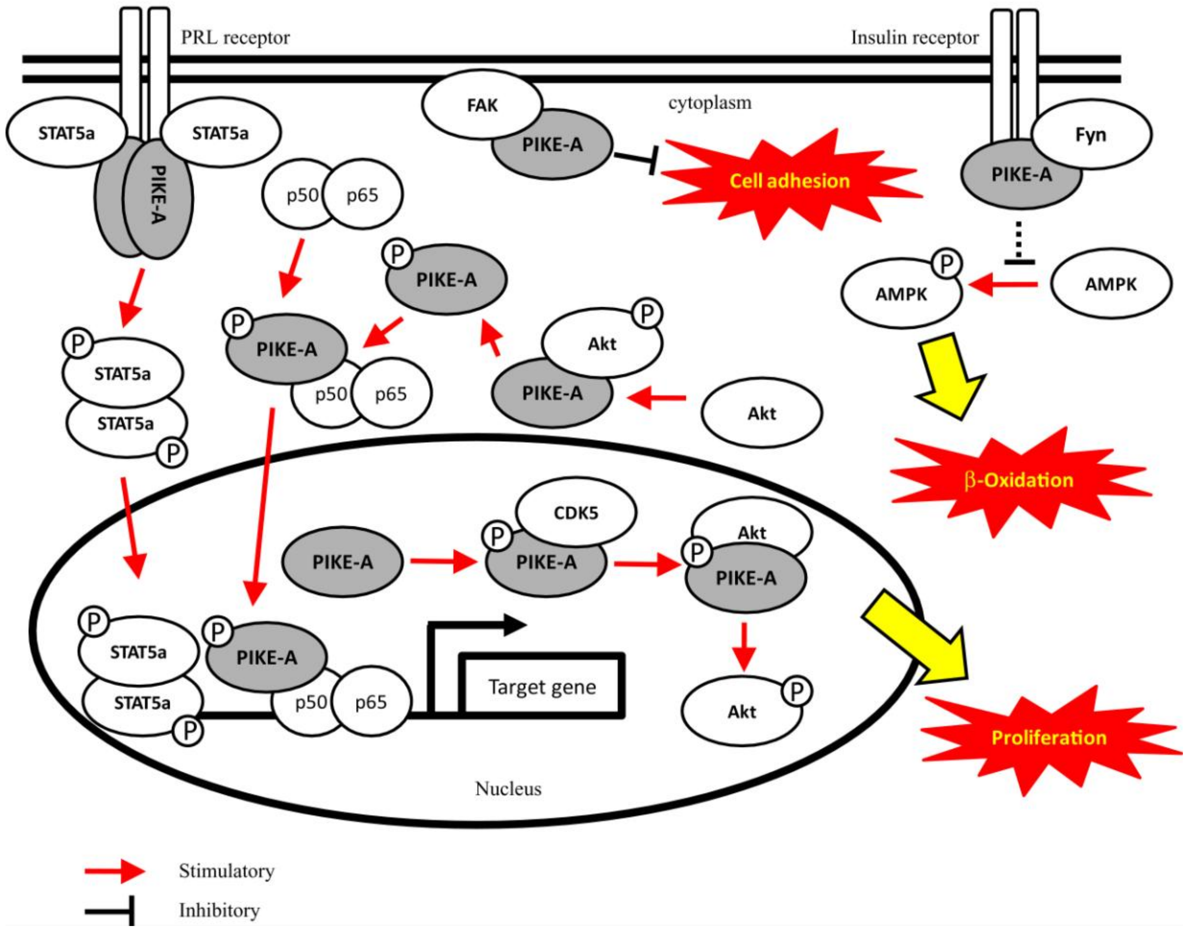

**FIGURE 2.** Summary of PIKE-A function in various physiological pathways.

(Fig. 2). Since disruption of focal adhesion is the prerequisite for cell migration[40], PIKE-A thus plays an essential role in such a process. As a matter of fact, U87MG glioblastoma cells with PIKE-A depletion exhibit more focal adhesion and are less sensitive to epidermal growth factor (EGF)–induced cell migration[39].

Recently, Cai et al. reported on a new signaling pathway of PIKE-A in mediating prostate cancer progression[41]. Similar to our observation in glioblastoma cells, the authors found that prostate cell lines with PIKE-A overexpression promoted cell proliferation in a GTPase-dependent manner. In addition to the classical role of PIKE-A in elevating the Akt activities, the authors found that PIKE-A interacts with the p50 subunit of transcription factor NF-κB through the GTPase domain and promotes its transcriptional activities (Fig. 2). NF-κB is an important transcription factor that displays abnormal activity in a variety of malignancies[42]. Consequently, the expression of NF-κB–responsive genes that are involved in cell proliferation, such as cyclin D1[43] and E-selectin[44], is enhanced in PIKE-A overexpressed cells. Therefore, PIKE-A mediates a cross-talk of the two critical pathways, Akt and NF-κB, in prostate cancer cell progression.

## REGULATION OF PIKE ACTIVITY

GTPase proteins are regarded as the molecular switch to trigger various signaling cascades[45]. In most cases, the GTPase could be activated by association with guanine nucleotide exchange factors (GEF), which induces a conformational change of GTPase and promotes the exchange of GDP to GTP. As a result, the affinity for the GTPase towards its specific effector is increased, leading to the initiation of a particular signaling pathway[46]. Similar to other GTPases, PIKE proteins have their specific GEF. In an attempt to search for the PIKE GEF, we performed a series of immunoprecipitations and found that phospholipase C gamma 1(PLC-γ1) interacts with the N-terminal proline-rich region of PIKE-S through its SH3 domain. Association of PLC-γ1 with PIKE-S markedly augments the binding of GTP and accelerates the GDP dissociation, thus increasing the activity of PIKE-S–bound PI3K[47] (Fig. 1). It is well recognized that PLC-γ1 is involved in the mitogenic function of growth factors in a lipase-independent manner[48]. However, how this function is executed is unknown, but it is hypothesized that the SH3 domain of PLC-γ1 triggers the activation of a then unidentified pathway[49]. Our demonstration that PLC-γ1 activates PIKE-S and the associated PI3K in the nucleus thus provides a mechanism for its mitogenic activity.

GTPase activity of PIKE can also be regulated by protein phosphorylation. In a study on the role of cyclin-dependent kinase 5 (cdk5) in glioblastoma cell proliferation, PIKE-A was found to be phosphorylated by the Cdk5 kinase on $Ser^{279}$[50] (Fig. 2). This phosphorylation is necessary to maintain the GTPase activity of PIKE-A, as well as its ability to activate Akt. Moreover, the cdk5-mediated phosphorylation of PIKE-A functions primarily within the nucleus to regulate the nuclear Akt activity. It has also been reported that Akt is a regulator to PIKE-A GTPase activity[41]. Akt phosphorylates PIKE-A, which is essential for maintaining its GTPase activity (Fig. 2). This phosphorylation is also critical for the interaction of PIKE-A and NF-κB to promote prostate cancer progression[41]. Conceivably, PIKE-A associates with Akt in an interactive manner in that the two proteins modulate the enzymatic activities of their partners mutually.

The PH domain is a structural motif present in a wide variety of proteins that bind phosphatidylinositol lipids within biological membranes[51,52]. Hu et al. have shown that the PH domain of PIKE plays a regulatory role to their cellular localization and GTPase activity[53]. Binding of phospholipids to the PIKE PH domain increases the GTPase activity. Interestingly, the PH domains in different PIKE members display differential affinity towards various phospholipids: PIKE-S PH showed no binding activity towards all the phospholipids probably because of the truncated structure; PIKE-L PH favors PI-3,4,5-$P_3$ rather than PI-3,5-$P_3$ and PI-3,4-$P_3$, whereas PIKE-A PH bound strongly to all phopholidipids tested with no distinguishable differences. It is suggested that the phospholipid/PIKE interaction causes a conformational change in the protein, rendering the C-terminal Arf-GAP domain accessible to its N-terminal domain, thus accelerating the GTPase activity. The proposed model is supported by the finding that the enzymatic activity of PIKE-A GTPase domain is low, if the Arf-GAP domain is deleted[31]. Therefore, intramolecular interaction between the GTPase domain and the C-terminal Arf-GAP domain results in high GTPase activity in PIKE. A recent structural study on the PIKE PH domain also suggests that the motif is responsible for binding the head groups of phosphoinositides in the cell membrane[54]. The authors show that the PH motif is critical for subcellular localization of PIKE, in which deletion of the putative nucleus localization signal (NLS) in this region eliminates its nuclear residency. Intriguingly, mutating the phospholipid-binding activity of the PIKE PH domain significantly reduces the membrane binding and triggers its nuclear translocation. Conceivably, the cellular localization is controlled by the interaction between phospholipids and the PIKE PH domain: rise of phospholipid concentration at the cytoplasmic membrane favors the membrane localization of PIKE, whereas depletion of membrane phospholipid binding shifts the protein into the nucleus.

## CELLULAR FUNCTIONS OF PIKE

Although the role of PIKE in cell survival and proliferation is well established, it is also involved in cellular activities other than prosurvival. The studies by Nie et al. suggest that PIKE-A is a GTPaseactivating protein (GAP) of small GTPase Arf1 and Arf6[55]. Moreover, they have shown that PIKE-A interacts with adaptor protein AP-1 and alters its cellular distribution. In cells overexpressing PIKE-A, AP-1 is redistributed from the perinuclear region to punctuate structures throughout the cell. Importantly, PIKE-A overexpression disrupts the cellular compartment responsible for receptor recycling, which is composed of AP-1, Rab4, and transferrin. Although the authors did not provide any specific molecular mechanism by which PIKE-A affected the AP-1/Rab4 compartment or any functional consequences of the compartment disorganization, their study clearly demonstrated that PIKE-A is involved in the intracellular cargo transportation between organelles.

The nuclear-specific PIKE-S also possesses the ability to facilitate redistribution of proteins. In cells without PIKE-S expression, BRAG2 (Brefeldin Reisistant Arf GEF 2) has a diffuse nuclear distribution. Ectopic expression of PIKE-S in HeLa cells induces the accumulation of coilin, the Cajal Body marker, in nucleoli[56]. BRAG2 is the GEF of Arf6, which localizes in the early endosome to regulate integrin internalization[57,58], yet its role in the nucleus remains largely unknown. The functional outcome of PIKE-S–mediated BRAG2 relocation has not been clearly defined. Presumably, PIKE-S overexpression may release BRAG2 from its anchoring site and trigger its accumulation in nucleoli where BRAG2 modulates the structure of nucleoli by initiating the change of proteins composition and regulating the ribonucleoprotein (RNP) biogenesis[56].

## FUNCTIONS OF PIKE IN PERIPHERIAL TISSUES

Although most of our understandings of PIKE functions are derived from the studies using neuron or brain cancer models, the broad expression profile of PIKE-A in different tissues has raised a fundamental question about the roles of PIKE-A in these peripheral tissues. To search for the physiological functions of PIKE, particularly those in the non-nervous system, we have generated the whole-body *PIKE*$^{-/-}$ mice using the LoxP/Cre system[59]. The knock-out mice with ablation of all PIKE isoforms are viable, fertile, and with no overt morphological defects, suggesting that PIKE is dispensable for embryonic development. However, we observed a significant reduction of white adipose tissue (WAT) mass in *PIKE*$^{-/-}$ mice, indicating that PIKE-A might be involved in regulating obesity development[60]. Studies using a genome-wide linkage scan revealed that the human chromosome trait 12q14.1, where the *CENTG1* gene locates, has a strong correlation with serum lipid level and energy intake, which further supports the notion that PIKE may be a potential factor in regulating body weight[61,62]. Indeed, *PIKE*$^{-/-}$ mice are resistant to diet-induced obesity and the associated diabetes[60], which strongly suggests that PIKE is a critical factor in controlling obesity progression. We have also shown that PIKE-A interacts with the insulin receptor and mediates its suppressive effect on AMP-activated protein kinase (AMPK) activation[60] (Fig. 2). AMPK is the master sensor of cellular energy status[63]. Activation of AMPK thus promotes a catabolic response, such as lipid oxidation, to provide extra energy. It is interesting to note that *PIKE*$^{-/-}$ muscle and fat have high AMPK phosphorylation and lipid oxidation, which might provide a mechanistic explanation to the slim phenotype of the knock-out mice[60]. Studies including the use of tissue-specific knock-out mice are now in progress in order to further delineate the primary action site of PIKE in triggering obesity development.

In addition, we observed a high death rate of pups from *PIKE*$^{-/-}$ dams, which died within 48 h after delivery[64]. When the newborns were fostered by wild-type dams, however, the pups were able to grow healthily. Further analysis of the mammary system of female *PIKE*$^{-/-}$ mice shows that the mammary tissue lacking PIKE-A expression fails to proliferate during lactation, indicating PIKE-A is critical for proliferation of normal tissues, particularly mammary glands, under physiological context. Collectively, our data suggest that PIKE-A might represent a novel signaling component of the prolactin (PRL)/STAT5 signaling, which couples STAT5a to the PRL receptor and meditates JAK2-dependent STAT5a phosphorylation[64] (Fig. 2). This finding further broadens the biological significance of PIKE-A as PRL is involved in a great variety of physiological functions ranging from metabolism to behavioral responses[65].

## CONCLUSION

Since the identification of PIKE-S as a mitogenic factor by enhancing nuclear PI3K activity, our understanding towards the physiological functions of PIKE proteins has been improved significantly during the last decade. It is now clear that PIKE interacts with numerous proteins/molecules to trigger multiple physiological functions in addition to maintaining cell survival (Table 1). In particular, the availability of PIKE knock-out mice generated by our laboratory has provided a powerful *in vivo* model to study the functions of various PIKE isoforms. From these reverse genetic studies, it was further confirmed that PIKE GTPases interact with a variety of signaling molecules in addition to PI3K and Akt. Yet, the precise role of PIKE in promoting different physiological outcomes, such as obesity development, remains to be specified. The generation of tissue-specific knock-out mice as well as isoform-specific knock-out mice would thus be necessary if a more detailed functional mapping of PIKE is needed.

**TABLE 1**
**Interaction Partners of PIKE and Their Functional Consequences after Association**

| PIKE Isoforms | Interaction Partner | Functions |
|---|---|---|
| PIKE-S | PI3K | Enhances the kinase activity of nuclear PI3K |
| | PLCγ | Enhances PIKE-S GTPase activity |
| | 4.1N | Abolishes PIKE-S/PI3K association |
| | BRAG2 | Accumulation of BRAG2 in nucleoli |
| PIKE-L | PI3K | Enhances the kinase activity of cytosolic PI3K |
| | Homer 1c | Activates PI3K by quisqualate |
| | UNC5B | Activates PI3K by netrin-1 |
| | SET | Prevents SET from proteolytic cleavage by AEP |
| PIKE-A | Akt | Potentiates the kinase activity of Akt and GTPase activity of PIKE-A |
| | Arf1 | Enhances GTPase activity of Arf1 |
| | AP1 | Inhibits the GAP activity of PIKE-A<br>Alters the intracellular distribution of AP1 |
| | NF-κB | Increases the transcriptional activity of NF-κB |
| | FAK | Augments the kinase activity of FAK |
| | Cdk5 | Enhances the GTPase activity of PIKE-A |
| | Insulin receptor | Inhibits of AMPK phosphorylation |
| | STAT5a | Enhances prolactin-induced STAT5a phosphorylation |

## ACKNOWLEDGMENT

This work is supported by grant from NIH RO1 (NS060608) to K.Ye.

## References

1. Chan, C.B. and Ye, K. (2007) PIKE GTPase are phosphoinositide-3-kinase enhancers, suppressing programmed cell death. *J Cell. Mol. Med.* **11,** 39–53.
2. Ye, K., Hurt, K.J., Wu, F.Y., Fang, M., Luo, H.R., Hong, J.J., Blackshaw, S., Ferris, C.D., and Snyder, S.H. (2000) Pike. A nuclear gtpase that enhances PI3kinase activity and is regulated by protein 4.1N. *Cell* **103,** 919–930.

3. Rong, R., Ahn, J.Y., Huang, H., Nagata, E., Kalman, D., Kapp, J.A., Tu, J., Worley, P.F., Snyder, S.H., and Ye, K. (2003) PI3 kinase enhancer-Homer complex couples mGluRI to PI3 kinase, preventing neuronal apoptosis. *Nat. Neurosci*. **6,** 1153–1161.
4. Ahn, J.Y., Hu, Y., Kroll, T.G., Allard, P., and Ye, K. (2004) PIKE-A is amplified in human cancers and prevents apoptosis by up-regulating Akt. *Proc. Natl. Acad. Sci. U. S. A.* **101,** 6993–6998.
5. Ahn, J.Y., Rong, R., Kroll, T.G., Van Meir, E.G., Snyder, S.H., and Ye, K. (2004) PIKE (phosphatidylinositol 3-kinase enhancer)-A GTPase stimulates Akt activity and mediates cellular invasion. *J. Biol. Chem.* **279,** 16441–16451.
6. Wymann, M.P. and Schneiter, R. (2008) Lipid signalling in disease. *Nat. Rev. Mol. Cell Biol.* **9,** 162–176.
7. Katso, R., Okkenhaug, K., Ahmadi, K., White, S., Timms, J., and Waterfield, M.D. (2001) Cellular function of phosphoinositide 3-kinases: implications for development, homeostasis, and cancer. *Annu. Rev. Cell Dev. Biol.* **17,** 615–675.
8. Ahn, J.Y., Rong, R., Liu, X., and Ye, K. (2004) PIKE/nuclear PI 3-kinase signaling mediates the antiapoptotic actions of NGF in the nucleus. *EMBO J.* **23,** 3995–4006.
9. Martelli, A.M., Cocco, L., Capitani, S., Miscia, S., Papa, S., and Manzoli, F.A. (2007) Nuclear phosphatidylinositol 3,4,5-trisphosphate, phosphatidylinositol 3-kinase, Akt, and PTen: emerging key regulators of anti-apoptotic signaling and carcinogenesis. *Eur. J. Histochem.* **51(Suppl 1),** 125–131.
10. Neri, L.M., Martelli, A.M., Borgatti, P., Colamussi, M.L., Marchisio, M., and Capitani, S. (1999) Increase in nuclear phosphatidylinositol 3-kinase activity and phosphatidylinositol (3,4,5) trisphosphate synthesis precede PKC-zeta translocation to the nucleus of NGF-treated PC12 cells. *FASEB J.* **13,** 2299–2310.
11. Divecha, N., Banfic, H., and Irvine, R.F. (1991) The polyphosphoinositide cycle exists in the nuclei of Swiss 3T3 cells under the control of a receptor (for IGF-I) in the plasma membrane, and stimulation of the cycle increases nuclear diacylglycerol and apparently induces translocation of protein kinase C to the nucleus. *EMBO J.* **10,** 3207–3214.
12. Yan, G.Z. and Ziff, E.B. (1995) NGF regulates the PC12 cell cycle machinery through specific inhibition of the Cdk kinases and induction of cyclin D1. *J. Neurosci.* **15,** 6200–6212.
13. Diakowski, W., Grzybek, M., and Sikorski, A.F. (2006) Protein 4.1, a component of the erythrocyte membrane skeleton and its related homologue proteins forming the protein 4.1/FERM superfamily. *Folia Histochem. Cytobiol.* **44,** 231–248.
14. Ye, K., Compton, D.A., Lai, M.M., Walensky, L.D., and Snyder, S.H. (1999) Protein 4.1N binding to nuclear mitotic apparatus protein in PC12 cells mediates the antiproliferative actions of nerve growth factor. *J. Neurosci.* **19,** 10747–10756.
15. Foa, L. and Gasperini, R. (2009) Developmental roles for Homer: more than just a pretty scaffold. *J. Neurochem.* **108,** 1–10.
16. Xiao, B., Tu, J.C., Petralia, R.S., Yuan, J.P., Doan, A., Breder, C.D., Ruggiero, A., Lanahan, A.A., Wenthold, R.J., and Worley, P.F. (1998) Homer regulates the association of group 1 metabotropic glutamate receptors with multivalent complexes of homer-related, synaptic proteins. *Neuron* **21,** 707–716.
17. Copani, A., Bruno, V.M., Barresi, V., Battaglia, G., Condorelli, D.F., and Nicoletti, F. (1995) Activation of metabotropic glutamate receptors prevents neuronal apoptosis in culture. *J. Neurochem*. **64,** 101–108.
18. Franke, T.F., Kaplan, D.R., and Cantley, L.C. (1997) PI3K: downstream AKTion blocks apoptosis. *Cell* **88,** 435–437.
19. Tang, X., Jang, S.W., Okada, M., Chan, C.B., Feng, Y., Liu, Y., Luo, S.W., Hong, Y., Rama, N., Xiong, W.C., Mehlen, P., and Ye, K. (2008) Netrin-1 mediates neuronal survival through PIKE-L interaction with the dependence receptor UNC5B. *Nat. Cell Biol.* **10,** 698–706.
20. Mehlen, P. and Furne, C. (2005) Netrin-1: when a neuronal guidance cue turns out to be a regulator of tumorigenesis. *Cell. Mol. Life Sci.* **62,** 2599–2616.
21. Keino-Masu, K., Masu, M., Hinck, L., Leonardo, E.D., Chan, S.S., Culotti, J.G., and Tessier-Lavigne, M. (1996) Deleted in colorectal cancer (DCC) encodes a netrin receptor. *Cell* **87,** 175–185.
22. Leonardo, E.D., Hinck, L., Masu, M., Keino-Masu, K., Ackerman, S.L., and Tessier-Lavigne, M. (1997) Vertebrate homologues of C. elegans UNC-5 are candidate netrin receptors. *Nature* **386,** 833–838.
23. Ming, G.L., Song, H.J., Berninger, B., Holt, C.E., Tessier-Lavigne, M., and Poo, M.M. (1997) cAMP-dependent growth cone guidance by netrin-1. *Neuron* **19,** 1225–1235.
24. Forcet, C., Stein, E., Pays, L., Corset, V., Llambi, F., Tessier-Lavigne, M., and Mehlen, P. (2002) Netrin-1-mediated axon outgrowth requires deleted in colorectal cancer-dependent MAPK activation. *Nature* **417,** 443–447.
25. Liu, G., Beggs, H., Jurgensen, C., Park, H.T., Tang, H., Gorski, J., Jones, K.R., Reichardt, L.F., Wu, J., and Rao, Y. (2004) Netrin requires focal adhesion kinase and Src family kinases for axon outgrowth and attraction. *Nat. Neurosci.* **7,** 1222–1232.
26. Ming, G., Song, H., Berninger, B., Inagaki, N., Tessier-Lavigne, M., and Poo, M. (1999) Phospholipase C-gamma and phosphoinositide 3-kinase mediate cytoplasmic signaling in nerve growth cone guidance. *Neuron* **23,** 139–148.
27. Liu, Z., Jang, S.W., Liu, X., Cheng, D., Peng, J., Yepes, M., Li, X.J., Matthews, S., Watts, C., Asano, M., Hara-Nishimura, I., Luo, H.R., and Ye, K. (2008) Neuroprotective actions of PIKE-L by inhibition of SET proteolytic degradation by asparagine endopeptidase. *Mol. Cell* **29,** 665–678.

28. Fan, Z., Beresford, P.J., Oh, D.Y., Zhang, D., and Lieberman, J. (2003) Tumor suppressor NM23-H1 is a granzyme A-activated DNase during CTL-mediated apoptosis, and the nucleosome assembly protein SET is its inhibitor. *Cell* **112,** 659–672.
29. Elkahloun, A.G., Krizman, D.B., Wang, Z., Hofmann, T.A., Roe, B., and Meltzer, P.S. (1997) Transcript mapping in a 46-kb sequenced region at the core of 12q13.3 amplification in human cancers. *Genomics* **42,** 295–301.
30. Nagase, T., Seki, N., Ishikawa, K., Tanaka, A., and Nomura, N. (1996) Prediction of the coding sequences of unidentified human genes. V. The coding sequences of 40 new genes (KIAA0161-KIAA0200) deduced by analysis of cDNA clones from human cell line KG-1. *DNA Res.* **3,** 17–24.
31. Xia, C., Ma, W., Stafford, L.J., Liu, C., Gong, L., Martin, J.F., and Liu, M. (2003) GGAPs, a new family of bifunctional GTP-binding and GTPase-activating proteins. *Mol. Cell. Biol.* **23,** 2476–2488.
32. Liu, X., Hu, Y., Hao, C., Rempel, S.A., and Ye, K. (2007) PIKE-A is a proto-oncogene promoting cell growth, transformation and invasion. *Oncogene* **26,** 4918–4927.
33. Su, W.T., Alaminos, M., Mora, J., Cheung, N.K., La Quaglia, M.P., and Gerald, W.L. (2004) Positional gene expression analysis identifies 12q overexpression and amplification in a subset of neuroblastomas. *Cancer Genet. Cytogenet.* **154,** 131–137.
34. Reifenberger, G., Ichimura, K., Reifenberger, J., Elkahloun, A.G., Meltzer, P.S., and Collins, V.P. (1996) Refined mapping of 12q13-q15 amplicons in human malignant gliomas suggests CDK4/SAS and MDM2 as independent amplification targets. *Cancer Res.* **56,** 5141–5145.
35. Reifenberger, G., Reifenberger, J., Ichimura, K., Meltzer, P.S., and Collins, V.P. (1994) Amplification of multiple genes from chromosomal region 12q13-14 in human malignant gliomas: preliminary mapping of the amplicons shows preferential involvement of CDK4, SAS, and MDM2. *Cancer Res.* **54,** 4299–4303.
36. Knobbe, C.B., Trampe-Kieslich, A., and Reifenberger, G. (2005) Genetic alteration and expression of the phosphoinositol-3-kinase/Akt pathway genes PIK3CA and PIKE in human glioblastomas. *Neuropathol. Appl. Neurobiol.* **31,** 486–490.
37. Stokoe, D., Stephens, L.R., Copeland, T., Gaffney, P.R., Reese, C.B., Painter, G.F., Holmes, A.B., McCormick, F., and Hawkins, P.T. (1997) Dual role of phosphatidylinositol-3,4,5-trisphosphate in the activation of protein kinase B. *Science* **277,** 567–570.
38. Meier, R., Alessi, D.R., Cron, P., Andjelkovic, M., and Hemmings, B.A. (1997) Mitogenic activation, phosphorylation, and nuclear translocation of protein kinase Bbeta. *J. Biol. Chem.* **272,** 30491–30497.
39. Zhu, Y., Wu, Y., Kim, J.I., Wang, Z., Daaka, Y., and Nie, Z. (2009) Arf GTPase-activating protein AGAP2 regulates focal adhesion kinase activity and focal adhesion remodeling. *J. Biol. Chem*. **284,** 13489–13496.
40. Lock, J.G., Wehrle-Haller, B., and Stromblad, S. (2008) Cell-matrix adhesion complexes: master control machinery of cell migration. *Semin. Cancer Biol.* **18,** 65–76.
41. Cai, Y., Wang, J., Li, R., Ayala, G., Ittmann, M., and Liu, M. (2009) GGAP2/PIKE-a directly activates both the Akt and nuclear factor-kappaB pathways and promotes prostate cancer progression. *Cancer Res.* **69,** 819–827.
42. Shen, H.M. and Tergaonkar, V. (2009) NFkappaB signaling in carcinogenesis and as a potential molecular target for cancer therapy. *Apoptosis* **14,** 348–363.
43. Hinz, M., Krappmann, D., Eichten, A., Heder, A., Scheidereit, C., and Strauss, M. (1999) NF-kappaB function in growth control: regulation of cyclin D1 expression and G0/G1-to-S-phase transition. *Mol. Cell. Biol.* **19,** 2690–2698.
44. Hallahan, D.E., Virudachalam, S., and Kuchibhotla, J. (1998) Nuclear factor kappaB dominant negative genetic constructs inhibit X-ray induction of cell adhesion molecules in the vascular endothelium. *Cancer Res.* **58,** 5484–5488.
45. Takai, Y., Sasaki, T., and Matozaki, T. (2001) Small GTP-binding proteins. *Physiol. Rev.* **81,** 153–208.
46. Rossman, K.L., Der, C.J., and Sondek, J. (2005) GEF means go: turning on RHO GTPases with guanine nucleotide-exchange factors. *Nat. Rev. Mol. Cell Biol.* **6,** 167–180.
47. Ye, K., Aghdasi, B., Luo, H.R., Moriarity, J.L., Wu, F.Y., Hong, J.J., Hurt, K.J., Bae, S.S., Suh, P.G., and Snyder, S.H. (2002) Phospholipase C gamma 1 is a physiological guanine nucleotide exchange factor for the nuclear GTPase PIKE. *Nature* **415,** 541–544.
48. Smith, M.R., Liu, Y.L., Matthews, N.T., Rhee, S.G., Sung, W.K., and Kung, H.F. (1994) Phospholipase C-gamma 1 can induce DNA synthesis by a mechanism independent of its lipase activity. *Proc. Natl. Acad. Sci. U. S. A.* **91,** 6554–6558.
49. Smith, M.R., Liu, Y.L., Kim, S.R., Bae, Y.S., Kim, C.G., Kwon, K.S., Rhee, S.G., and Kung, H.F. (1996) PLC gamma 1 Src homology domain induces mitogenesis in quiescent NIH 3T3 fibroblasts. *Biochem. Biophys. Res. Commun.* **222,** 186–193.
50. Liu, R., Tian, B., Gearing, M., Hunter, S., Ye, K., and Mao, Z. (2008) Cdk5-mediated regulation of the PIKE-A-Akt pathway and glioblastoma cell invasion. *Proc. Natl. Acad. Sci. U. S. A.* **105,** 7570–7575.
51. Lemmon, M.A. (2007) Pleckstrin homology (PH) domains and phosphoinositides. *Biochem. Soc. Symp*. **(74),** 81–93.
52. Lemmon, M.A. (2004) Pleckstrin homology domains: not just for phosphoinositides. *Biochem. Soc. Trans.* **32,** 707–711.
53. Hu, Y., Liu, Z., and Ye, K. (2005) Phosphoinositol lipids bind to phosphatidylinositol 3 (PI3)-kinase enhancer GTPase and mediate its stimulatory effect on PI3-kinase and Akt signalings. *Proc. Natl. Acad. Sci. U. S. A.* **102,** 16853–16858.

54. Yan, J., Wen, W., Chan, L.N., and Zhang, M. (2008) Split pleckstrin homology domain-mediated cytoplasmic-nuclear localization of PI3-kinase enhancer GTPase. *J. Mol. Biol.* **378,** 425–435.
55. Nie, Z., Fei, J., Premont, R.T., and Randazzo, P.A. (2005) The Arf GAPs AGAP1 and AGAP2 distinguish between the adaptor protein complexes AP-1 and AP-3. *J. Cell Sci.* **118,** 3555–3566.
56. Dunphy, J.L., Ye, K., and Casanova, J.E. (2007) Nuclear functions of the Arf guanine nucleotide exchange factor BRAG2. *Traffic* **8,** 661–672.
57. Dunphy, J.L., Moravec, R., Ly, K., Lasell, T.K., Melancon, P., and Casanova, J.E. (2006) The Arf6 GEF GEP100/BRAG2 regulates cell adhesion by controlling endocytosis of beta1 integrins. *Curr. Biol.* **16,** 315–320.
58. Hiroi, T., Someya, A., Thompson, W., Moss, J., and Vaughan, M. (2006) GEP100/BRAG2: activator of ADP-ribosylation factor 6 for regulation of cell adhesion and actin cytoskeleton via E-cadherin and alpha-catenin. *Proc. Natl. Acad. Sci. U. S. A.* **103,** 10672–10677.
59. Bucholtz, F. (2008) Principles of site-specific recombinase (SSR) technology. *J. Vis. Exp.* **(15)**, pii: 718
60. Chan, C.B., Liu, X., Jung, D.Y., Jun, J.Y., Luo, H.R., Kim, J.K., and Ye, K. (2010) Deficiency of PIKE protects mice from diet-induced obesity and insulin resistance. *Diabetes* [Epub ahead of print]
61. Collaku, A., Rankinen, T., Rice, T., Leon, A.S., Rao, D.C., Skinner, J.S., Wilmore, J.H., and Bouchard, C. (2004) A genome-wide linkage scan for dietary energy and nutrient intakes: the Health, Risk Factors, Exercise Training, and Genetics (HERITAGE) Family Study. *Am. J. Clin. Nutr.* **79,** 881–886.
62. Bosse, Y., Chagnon, Y.C., Despres, J.P., Rice, T., Rao, D.C., Bouchard, C., Perusse, L., and Vohl, M.C. (2004) Genome-wide linkage scan reveals multiple susceptibility loci influencing lipid and lipoprotein levels in the Quebec Family Study. *J. Lipid Res.* **45,** 419–426.
63. Towler, M.C. and Hardie, D.G. (2007) AMP-activated protein kinase in metabolic control and insulin signaling. *Circ. Res.* **100,** 328–341.
64. Chan, C.B., Liu, X., Ensslin, M.A., Dillehay, D.A., Ormandy, C.J., Son, P., Serra, R., and Ye, K. PIKE-A is required for prolactin-mediated STAT5a activation in mammary gland development. *EMBO J.* **29(5),** 956–968.
65. Freeman, M.E., Kanyicska, B., Lerant, A., and Nagy, G. (2000) Prolactin: structure, function, and regulation of secretion. *Physiol. Rev*. **80,** 1523–1631.
